# Association of genotype III of dengue virus serotype 3 with disease outbreak in Eastern Sudan, 2019

**DOI:** 10.1186/s12985-020-01389-9

**Published:** 2020-07-30

**Authors:** Mawahib H. Eldigail, Hazem A. Abubaker, Fatima A. Khalid, Tajeldin M. Abdallah, Hassan H. Musa, Mohamed E. Ahmed, Gamal K. Adam, Mustafa I. Elbashir, Imadeldin E. Aradaib

**Affiliations:** 1 Molecular Biology Laboratory, Faculty of Veterinary Medicine, University of Khartoum, P.O. Box 32, Khartoum North, Sudan; 2 Department of Internal Medicine and Microbiology, Faculty of Medicine, University of Kassala, El-Gadarif, Sudan; 3 Department of Medical Microbiology, Faculty of Medical laboratory Sciences, University of Khartoum, El-Gadarif, Sudan; 4 Zamzam Unit for Medical Research (ZUMR), Vectore Borne and Zoonotic Diseases Research Laboratory, Zamzam University College, Khartoum, Sudan; 5 Department of Microbiology, Faculty of Medicine, University of El-Gadarif, El-Gadarif, Sudan; 6 Department of Microbiology, Faculty of Medicine, University of Khartoum, El-Gadarif, Sudan

**Keywords:** Dengue virus, ELISA, RT-PCR; sequence analysis, Phylogeny, Sudan

## Abstract

**Background:**

Dengue fever (DF) is an arthropod-borne disease caused by dengue virus (DENV). DENV is a member of the genus *Flavivirus* in the family *Flaviviridae*. Recently, DENV has been reported as an important emerging infectious viral pathogen in Sudan. Multiple outbreaks and sporadic cases of DF have been frequently reported in the eastern region of Sudan. The present study was conducted to confirm DENV outbreak in Kassala State, eastern Sudan, 2019, and to provide some information on the molecular characterization of the DENV isolate associated with the disease outbreak.

**Methods:**

A hundred serum samples were collected during the outbreak from residents of Kassala State, Sudan, 2019. ELISA was used to detect DENV non structural protein NS1 (DENV-NS1) in acute phase sera sampled during the disease outbreak. RT-PCR assays were used to amplify a fragment of the capsid/pre-membrane region (CprM) of the viral polyprotein gene. The PCR products of the amplified CprM region of the viral polyprotein gene were purified and partial sequences were generated and used to confirm the specificity of DENV sequences and to identify the virus serotype. Phylogenetic tree was constructed to determine the genotype of DENV associated with the outbreak.

**Results:**

Using DENV-NS1 ELISA assay, DENV infection was confirmed in 23% sampled sera. The detection of DENV RNA was made possible using group-specific RT-PCR assay. The virus was serotyped as DENV serotype 3 (DENV-3) using DENV serotype-specific RT-PCR assay. Phylogenetic analysis of the partial CprM sequences of the viral polyprotein gene indicates that the virus belonged to genotype III of DENV-3.

**Conclusion:**

The scientific data presented in this investigation confirmed that genotype III of DENV-3 was associated with the disease outbreak in eastern Sudan, 2019. The study represents the first report on molecular characterization of DENV-3 in Sudan.

## Backgroud

Dengue fever is a mosquito-borne viral disease that typically occurs in various areas of sub-Saharan Africa [1–5]. Dengue fever is caused by Dengue virus (DENV), which exists in four serologically distinct serotypes designated as (DENV-1), (DENV-2), (DENV-3) and DENV-4). The severity of DENV infection varies from mild fever to complicated clinical hemorrhagic disease leading to shock and subsequent death [6–8]. In the recent years, DENV has spread significantly to different States of Sudan [9–12]. Currently, DENV infection constitutes one of the major unresolved public health problems in the country. The major economic losses occur in areas of endemicity in the eastern part of Sudan particularly, Kassala and the Red Sea States [13–15]. In Sudan, most dengue fever cases are asymptomatic or probably accompanied by a mild febrile illness. However, severe cases are usually observed when more than one DENV serotypes are coexisting in a particular area of endemicity in the country [7, 8]. The first documented outbreak of DENV in Sudan occurred in 1986 among residents of the Red Sea State [16]. Shortly thereafter, several epidemic cycles of dengue have been recorded among residents of the neighboring Kassala State based on clinical presentation. Subsequently, serological evidence of DENV in Kassala State was demonstrated by detection of DENV immunoglobulin G (IgG) antibodies using ELISA assay [9, 11, 12]. Confirmation of active circulation of DENV in this State was made possible by direct detection of DENV immunoglobulin M (IgM) antibodies and reverse transcription RT-PCR [14, 17]. DENV serotypes 1 and 2 were reported in the Red Sea State whereas DENV-2 was reported as the prevalent serotype circulating in Kassala State [16, 17]. However, DENV serotype 3 (DENV-3) has never been reported in Kassala State, eastern Sudan. A recent sero-epidemiologic survey reported an exceptionally high prevalence (47.6%) of DENV-specific IgG antibodies in El-Gadarif State, eastern Sudan, suggesting considerable circulation of DENV in the area at some time in the past [18]. However, active circulation of DENV in El-Gadarif State is yet to be confirmed by conventional virus isolation attempts and molecular characterization studies. It is, therefore, becoming obvious that DENV has spread to different part of the country and that the disease becomes endemic in the eastern region of Sudan. It is possible that DENV spreads to other areas, including northward into Egypt and eastward to Ethiopia, Eretria, and probably across the Red Sea into Saudi Arabia. How DENV travels is unclear but probably involves movement of infected patients and mosquito vectors as well as intercontinental transfer of commercial products [19–24]. Recently, high incidence of dengue fever has been reported in eastern Sudan as witnessed by frequent sporadic cases and multiple outbreaks in 2010, 2013, 2017 and 2018 [17, 25]. The highest incidence of infections occurs between the months of September and November, which coincides with the high rainy season. A previous report has described the molecular characterization of DENV-2 in Kassala State, eastern Sudan [17]. With the exception of this report, no information is currently available in regard to the serotypes and associated genotypes of DENV circulating in Sudan. In this context, further study on molecular characterization of DENV isolates would be necessary to better understand the biology, ecology and the molecular epidemiology of the disease. In the present study, an outbreak of DENV serotype 3 (DENV-3) characterized by acute febrile illness occurred in Kassala State, eastern Sudan, 2019. ELISA NS1 assay was used to detect early infection in acute phase sera of infected patients. The detection of DENV and identification of the virus serotype was determined by serogroup-specific and serotype-specific DENV RT-PCR assays, respectively. The partial genome sequences generated from the amplified is Capsid/premembrane (CprM) protein gene were purifies and employed for subsequent phylogenetic analysis. Phylogenetic tree was constructed to determine the genotypes of the DENV serotype associated with the disease outbreak. The results of this study would be expected to provide invaluable clues for improved surveillance and control of the disease in Sudan.

## Methods

### The outbreak

On the 8th of August 2019, FMOH has declared an outbreak of dengue fever in Kassala State. The cases were first reported from Kassala State and then spread to other states. Currently 24 localities in nine states are affected by dengue, namely Kassala, North Darfur, Red Sea, South Darfur, West Darfur, EL-Gadarif, North Kordufan, Sennar and East Darfur. The majority of cases were reported from Kassala state and North Darfur. The clinical signs include fever, joint pain and headache [25]. In the present investigation, a wide range of symptoms were observed during clinical presentation of the suspect residents of Kassala State. The symptoms include high-grade fever, headache, and joint pain, with or without vomiting. Approximately 10% of the infected patients showed hemorrhagic manifestations. Since the start of the outbreak on the 8th of August 2019, dengue has spread to seven states across the country. The public health response measures have been implemented. A task force committee has been activated at federal level. Outbreak investigation teams at state and local levels have been reactivated, and all the reported cases have been investigated. Application of insecticides for control of the mosquito vector has been conducted in the State.

### Case definitions

A suspected DENV infected case was defined as high-grade fever of 38.5 °C with or without hemorrhagic manifestations, vomiting, headache, and joint pain. A confirmed DENV case was defined as laboratory-confirmed acute or recent DENV infection by positive DENV NS1 detection and/or positive reverse transcription PCR (RT-PCR).

### Specimen collection and preparation

Blood samples were collected from a total of 100 suspected DENV case-patients during the disease outbreak between September and December 2019. Blood samples were allowed to clot, and serum was separated for serologic diagnostic screening and RT-PCR amplification.

### Virus isolation

Virus detection and identification were almost exclusively based on detection of DENV NS1 by ELISA assay and identification of the viral genome by conventional RT-PCR amplification assay.

### Enzyme linked immunosrobent assay (ELISA)

The sample sera were screened for the detection of DENV- NS1 enzyme-Linked Immunosorbent Assay (ELISA). The ELISA assay was performed using a commercially available non-structural protein 1 (NS1) DENV ELISA Kit (Euroimmun AG, Luebeck, Germany), in accordance with the manufacturer’s specifications. Details of the methodology of the ELISA were described in a previous report [18].

### Viral nucleic acid extraction

The QIAamp extraction kit (QIAamp, Hamburg, Germany) was used to extract viral nucleic acids. RNAs were extracted from infected patient sera as per manufacturer’s instructions. Briefly, 140 μl of serum added to 560 μl AVL buffer containing carrier RNA into a 1.5 ml micro-centrifuge tube and mixed by pulse vortexing for 15 s. The mixture was incubated at room temperature for 10 min. 560 μl of absolute ethanol were added and mixed by pulse-vortexing for 15 s. 630 μl of the mixture were transferred to QIAamp spin column mounted on 2 ml collection tube and centrifuged at 5000×g for 1 min. The column was then transferred to another collection tube and the remaining 630 μl of the mixture was again spin at the same speed. The column was then washed twice by 500 μl of washing buffers WB1 andWB2, respectively. Finally, dsRNAs were carefully eluted by 60 μl of AVE buffer equilibrated to room temperature. RNAs were quantified using a spectrophotometer at 260 nmwave length. We quantify the samples to make sure that they contain adequate RNA before application of RT-PCR and subsequent sequencing. RNA extracts were then kept at − 20 °C until used for PCR amplification. Five μl of the nucleic acid were used for RT-PCR amplification.

### Serogroup-specific RT-PCR assay for detection DENV

The serogroup-specific RT-PCR assay for detection DENV RNA was performed targeting a 511 bp fragment of CprM gene of the the viral polyprotein gene. The RT-PCR assay was performed basically as described by Lanciotti et al., [26]. Briefly, DENV RNA was amplified with conventional RT-PCR by using a Super Script III One-Step RT-PCR System with Platinum Taq High Fidelity (Invitrogen, Carlsbad, CA, USA). Thermal profiles were performed on a Techne PHC-2 thermal cycler (Techne, Princeton, NJ,USA); reactions were incubated for 30 min at 50 °C, followed by 40 cycles of 95 °C for 1 min, 55 °C for 1 min, and72°C for 1 min, and a final incubation at 68 °C for 10 min. Following the amplification, 5 μl of the amplified PCR products were visualized on ethidium bromide-stained agarose gel under UV light.

### Serotype-specific RT-PCR assay for identification DENV

The semi-nested serotype-specific RT-PCR assay for identification DENV RNA was performed using the same forward primer D1 and an internal reverse primer T3 to amplify a 290- bp PCR product of the same CprM region of the viral polyprotein gene. The thermal cycling profiles for the serotype-specific RT-PCR assay was performed basically as described by Lanciotti et al. [26]. Details of the universal primers employed for amplification of DENV serogroup and serotype-specific PCR products are summarized in (Table 1).

**Table 1:** Oligoneucleotide primer sequences used for detection of dengue virus serogroup and identification of dengue virus serotypes using RT-PCR assays. The primer sequences and the expected PCR products are shown in the table as described by Lanciotti et al. 1992 26]

Primer	Sequence	PCR product
**D1**	5′-TCAATATGCTGAAACGCGCGAGAAACCG-3′	511 bp
**D2**	5′-TTGCACCAACAGTCAATGTCTTCAGGTTC-3′	
**TS1**	5′-CGTCTCAGTGATCCGGGGG-3′	482 (Dl and TS1) DENV-1
**TS2**	5′-CGCCACAAGGGCCATGAACAG-3′	119 (Dl and TS2) DENV-2
**TS3**	5′-TAACATCATCATGAGACAGAGC-3′	290 (Dl and TS3) DENV-3
**TS4**	5′-CTCTGTTGTCTTAAACAAGAGA-3′	392 (Dl and TS4) DENV-4

### Dengue virus partial-genome sequencing and phylogeny

Five serum samples from the outbreak were positive for DENV by conventional RT-PCR. The SuperScript III One-Step RT-PCR System with Platinum Taq High Fidelity enzyme mix in accordance with the manufacturer’s instructions by using segment-specific primers. The samples were cycled as follows: 50°Cfor 30 min; 94 °C for 2 min; 40 cycles at 94 °C for 1 min, 55 °C for for1 min, 68 °C for 1 min; and a final extension at 68 °C for 5 min. The 511-bp DENV serogroup-specific PCR products were purified using QIAquick PCR purification Kit (QIAgen, Germany) and sent for sequencing in a commercial company (Colors Lab, Cairo, Egypt). Resulted sequences were edited using BioEdit software and the Basic Local Alignment Search Tool (BLAST) of NCBI (National Center for Biotechnology Information, Bethesda, MD) and used to confirm the identity of the generated sequences in the GenBank nucleotide database.

### Phylogenetic analysis

The sequences were aligned using Molecular Evolutionary Genetics Analysis (MEGA) software version 7.0. The phylogenetic tree was constructed using the Maximum likelihood statistical method based on the Tamura-Nei model [27]. The GenBank accession numbers, the country of origin and the date of isolation were given for each virus isolate when available. Bootstrap analysis of 1000 replicates was applied and values were given at relevant nodes of the constructed tree.

## Results

### Suspected DENV case-patients

Serum samples from 100 persons whose illness fit the clinical case definition were tested by NS1 ELISA to detect early infection. The samples were also tested by RT-PCR to detect viral RNA during acute phase of the disease. Twenty three (23%) of these suspected case-patients were positive by ELISA NS1.

### Detection and identification of DENV using RT-PCR assay

The detection of DENV RNA was made possible using group-specific RT-PCR assay. Five serum samples from the outbreak were positive for DENV by conventional RT-PCR. A pair of universal primers (D1 and D2) was designed from the CprM region of the viral polyprotein gene and employed in PCR assay. The RT-PCR assay amplified the DENV-specific 511 bp PCR product from five (5.0%) of suspected cases. The detection of DENV serotype was made possible using serotype-specific RT-PCR assay. A second pair of primers consisting of the same forward primer D1 and a reverse primer T3 was used to amplify a 290 bp PCR product in a semi-nested format. The virus was serotyped as DENV serotype 3 (DENV-3) using Primers (D1 and T3), which amplified the expected 290 bp PCR product as described by by Lanciotti et al. 1992 [26].

### Sequence analysis and phylogenetic relationship

**Fig. 1:**
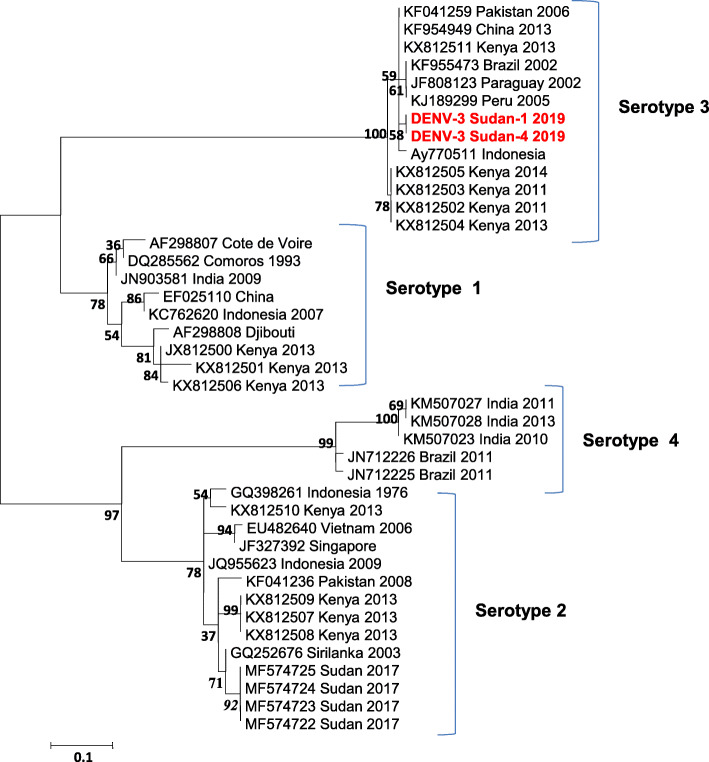
Phylogenetic tree based on serogroup-specific ENV CprM sequences of the viral polyprotein gene placed the Sudan DENV isolates with DENV serotype 3. The phylogenetic tree was constructed using the Maximum Likelihood method based on the Tamura-Nei model. Evolutionary analyses were conducted in MEGA7 with 1000 bootstrap replicates shown at relevant nodes of the constructed tree. The phylogenetic tree involved analysis of 41 nucleotide sequences of Known serotypes of DENV isolates circulating globally. The accession numbers, country of origin and date of isolation were mentioned for each virus isolate when available. The Sudan DENV-3 sequences recovered in this study were highlighted in red color for clarity. The scale bar expresses the genetic distance

**Fig. 2:**
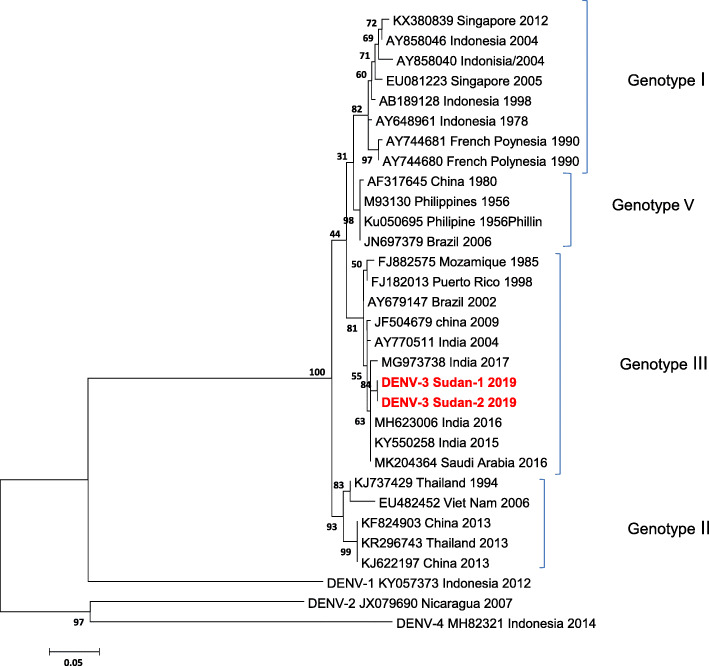
Phylogenetic tree based on DENV-3 CprM sequences of the viral polyprotein gene placed the Sudan DENV isolates with genotype 3 of DENV serotype 3. The phylogenetic tree was constructed using the Maximum Likelihood method based on the Tamura-Nei model. Evolutionary analyses were conducted in MEGA7 with 1000 bootstrap replicates shown at relevant nodes of the constructed tree. A total of 31sequences of DENV-3 isolates available in NCBI GenBank were used to determine the genotype of the DENV-3 isolates employed in this study. The accession numbers, country of origin and date of isolation were mentioned for each virus isolate when available. The constructed phylogenetic tree placed the viral sequences recovered in this study with genotype III genetic lineage of DENV-3. The Sudan viral sequences are highlighted in red color for clarity. The scale bar expresses the genetic distance

The primary 511-bp PCR products were purified using QIAquick PCR purification kit (Felden, Germany). Bidirectional sequence fragments of the forward and reverse primers were generated for the selected samples. The sequences were edited manually to correct possible base calling errors using BIOEDIT 7.0 and were subsequently joined to reconstruct a fragment of the expected PCR product. The consensus sequences were aligned with the corresponding region of the viral polyprotein gene of known DENV serotypes circulating globally. The constructed phylogenetic tree, based on the partial sequences of the CprM region of the viral polyprotein gene of DENV serogroup placed the generated sequences with DENV serotype 3 (DENV-3) as illustrated in (Fig. 1). The sequence analysis showed that the 2 isolated sequences of DENV-3 (DENV-3 Sudan-1 and DENV-3 Sudan-2) are identified and showed 100% homology identical. The constructed phylogenetic tree, based on the partial sequences of the viral polyprotein gene of DENV-3, illustrated that the isolated DENV-3 sequences belonged to genotype III, with closed similarity to DENV-3 isolates circulating in India and Saudi Arabia (Fig. 2).

The partial CprM sequences of the polyprotein gene submitted to the GenBank under accession number MT012479.

## Discussion

Despite the fact that DENV is endemic in Africa, information documenting active circulation of DENV in Sudan is very scanty. DENV transmission among human population in Sudan was first recognized in 1986 when an outbreak of the disease occurred in the Red Sea State, eastern Sudan [16]. Currently, DENV is spreading widely to the western and central Sudan but the eastern part of the country is endemic with the disease [12, 28, 29]. The lack of rapid and accurate diagnosis and unavailability of detailed analysis of DENV outbreak in most African countries have resulted in limited information related to the true disease burden and economic impact of the disease [1, 27]. In Sudan, very little information is available about the circulating serotypes and associated genotypes. The serological surveys constitute the bulk of the research conducted on the epidemiology of the disease [11–14, 30, 31]. The virological and molecular characterization studies have not been investigated sufficiently to describe the serotypes and associated genotypes of DENV circulating in the country. DENV infection usually causes mild fever and may proceed to acute febrile illness leading to high morbidity. In some cases the disease may be complicated by dengue hemorrhagic fever (DHF) or Dengue shock syndrome (DSS) leading to death. In areas of endemicity of eastern Sudan, concurrent or previous infection with heterologous DENV serotype may result in complicated hemorrhagic manifestation leading to significant mortalities among the infected patients [6, 7, 32]. All four serotypes of DENV are actively circulating in African [1]. However, only three dengue virus serotypes (DENV-1, 2, 3) have been reported in Sudan [16, 17, 28–33]. In a previous report, DENV serotype 2 (DENV-2) was reported as an etiological agent of a disease outbreak in Kassala State, 2017. Phylogenetic analysis revealed that the isolated virus sequences belong to the cosmopolitan genotype of DENV-2 [17]. DENV-1 and DENV-3 have been reported in the Red Sea State and in Darfur region of western Sudan but no information is available about the molecular characterization of the isolated viruses. Infection with DENV-4 has never been reported in Sudan, but it has been documented in parts of Africa and in Europe from travelers returning from Africa [1, 34]. The eastern part of Sudan is bordered by the Red Sea, which provides a major sea port connecting the Asian and African continents. The incursion of DENV to Sudan is likely to occur through the sea port as it facilitates the international trade of goods and essential commodities from the Asian countries. The movement of the infected travelers between endemic and DENV-free areas would also play an important role in transmission and significant spread of the disease [20–23]. Clinical outcomes of dengue cases may be influenced by the circulation of multiple DENV serotypes and is considered a factor in the reemergence of dengue hemorrhagic fever. The previous report of the circulation of DENV-2 and the present report of DENV-3 in Kassala State would probably suggest co-circulation of both DENV-1 and DENV-2, which results in increased severity of the disease. However, further studies on co-circulation of multiple DENV serotypes and associated genotypes in this area of endimicity would be necessary to confirm this assumption. The environmental changes such as global warming, changes in land use and water management can have an important influence on the observed changes in disease caused by hemorrhagic fever viruses, and DENV is not an exception. It is well documented that DENV is endemic in Africa with outbreaks almost occurring as a result of favorable environmental conditions [1, 18]*.* In this context, the fast growing petroleum industry and the development of new irrigation projects and agriculture schemes in the country rendered the virus to become more adapted to different ecological systems. It is worth mentioning that the fall season in Sudan starts from August to November. Heavy rains were observed in Kassala State during those months of the year 2019. The heavy rains could be linked to the breeding of the mosquito vector in a large population density in the region, which coincided with the time of the outbreak onset. In such a situation, the mosquito control operations should be initiated to reduce the high density of the vector population. The limited information about the disease outbreak was attributed to the lack of appropriate high-containment facilities required for virus isolation. In addition, unavailability of appropriately sampled and stored acute-phase serum was believed to have negative impact on the detailed analysis of this outbreak.

In the present investigation, serum specimens from 23 patients were positive for DENV NS1 ELISA. However, only 5 of the suspected patients were positive for DENV RT-PCR. This is probably due to collection of most serum samples at a later stage after the appearance of DENV-specific Ig M/ IgG antibodies, which resulted in neutralization and subsequent clearance of the virus from the blood circulation. In this study, we have identified two sequences of genotype III of DENV-3, which showed 100% sequence homology. Thus, genotype III of DENV-3 was associated with the disease outbreak in Kassala State, 2019. DENV-3 has never been reported in the endemic area of Kassala State, eastern Sudan. This is the first molecular characterization study of DENV-3 in Sudan. Future studies on molecular characterization of DENV isolates would be advantageous to determine the serotypes and associated genotypes of DENV circulating in Sudan. The sequence analysis and phylogenetic studies would provide a better understanding regarding the spread and incursion of the virus in areas at risk for DENV in the country [18]. The finding that the genotype III of DENV-3 was associated with disease outbreak in Kassala State illustrates how different virus genotypes can move across continents, possibly by viremic travelers, infected mosquito vectors, or intercontinental transfer of commercial products [35–37]. In addition rapid urbanization and globalization is associated with the expansion of dengue transmission by providing a conducive environment for the mosquito vector [38–40]. It is, therefore, becoming obvious that genotype III of DENV-3 is now broadly distributed in Sudan, which is located in the east central Africa. The addition of DENV sequences from Sudan enhances our understanding of the detailed ecology, biology and the molecular epidemiology of the virus.

## Conclusion

The circulation of DENV-3 is reported in this study for the first time in Kassala State, eastern Sudan, 2019. The genotype III of DENV-3 was confirmed as the causative agent of the disease outbreak. Further studies should focus on molecular characterization of DENV isolates circulating in the country. The molecular characterization studies would provide invaluable tool to trace the movement of the virus in this region of the African continent. The frequent occurrence of sporadic cases and multiple DENV outbreaks necessitates the need for improved surveillance programs and prevention measures to control DENV infection in Sudan.

## Data Availability

Data and materials are available upon request from the corresponding author.

## Abbreviations

DEN: Dengue
DENV: Dengue virus
Ig G: Immunoglobulin G
Ig M: Immunoglobulin M
ELISA: Enzyme-linked immunosorbent assay
RT-PCR: Reverse transcriptase polymerase chain reaction
μl: Microliter
FMOH: Fedral Ministry of Health
CprM: Capsid/premembrane protein gene
